# Biological Control of Tomato Root Rot Caused by *Rhizoctonia solani* Using Microorganisms

**DOI:** 10.3390/jof12050313

**Published:** 2026-04-24

**Authors:** Zhan-Bin Sun, Xiao-Feng Li, Xin-Ran Pei, Xin-Pei Wang, Zheng Zhou, Han-Xu Pan, Man-Hong Sun

**Affiliations:** 1 School of Light Industry Science and Engineering, Beijing Technology and Business University, Beijing 100048, China; twins5616@126.com (Z.-B.S.);; 2 State Key Laboratory for Biology of Plant Diseases and Insect Pests, Institute of Plant Protection, Chinese Academy of Agricultural Sciences, Beijing 100193, China

**Keywords:** tomato root rot, *Rhizoctonia solani*, biocontrol bacteria, biocontrol fungi

## Abstract

*Rhizoctonia solani* is the main pathogen that causes tomato root rot, which is a soilborne disease that seriously affects tomato production, leading to huge economic losses. Biocontrol is an excellent control method for suppressing plant disease, as it is environmentally friendly, safe, and sustainable. Currently, reviews of the biocontrol of tomato root rot caused by *R. solani* are scarce. In this review, biocontrol agents, including bacteria and fungi, that can control tomato root rot caused by *R. solani* are discussed in depth, as well as their control effects. Moreover, this review systematically analyzes the potential control mechanisms of biocontrol agents, including the production of cell-wall-degrading enzymes, the production of metabolites, mycoparasitism, the induction of plant systemic resistance, and competition. Considerations for the practical application of biocontrol agents, including their formulation, reproducibility under field conditions, environmental variability, regulatory considerations for some microbial agents, and limitations, are also highlighted and discussed. Finally, further research suggestions are made for the future control of tomato root rot caused by *R. solani*. This review provides a basis for the field application of biocontrol agents to control tomato root rot caused by *R. solani*.

## 1. Introduction

Tomatoes (*Solanum lycopersicum*, belonging to Solanaceae) are economically important vegetable crops that are extensively cultivated and consumed worldwide, either fresh or after processing [1,2]. Tomatoes are rich in nutrients and bioactive compounds—such as vitamins A, C, and E; amino acids; ferulic acid; ascorbic acid; folate; minerals; lycopene; β-carotene; dietary fiber; flavonoids; and α-tomatine—which exert beneficial effects on human health through their antioxidant, anti-inflammatory, cardioprotective, and antimicrobial activities [3,4,5,6].

Numerous factors can affect the growth and production of tomatoes, including abiotic stresses such as temperature, water availability, light, and growing media [7,8,9,10]. Additionally, the diseases caused by bacteria, fungi, viruses, and nematodes are some of the most serious biotic stresses that can significantly influence the growth and production of tomatoes, causing huge economic and production losses. Common tomato diseases mainly consist of bacterial diseases, such as bacterial spot, wilt, speck, and canker [11,12,13]; fungal diseases, such as root rot, gray mold, early blight, late blight, Fusarium wilt, and Verticillium wilt; nematode diseases, such as root-knot nematodes; and viral diseases, such as spotted wilt, Pepino mosaic virus, and yellow leaf curl virus [14,15,16,17,18,19,20,21,22,23,24]. Among these plant diseases, root rot diseases are a significant concern because of the threat they pose to tomato productivity worldwide.

Tomato root rot diseases can be caused by different types of fungal plant pathogens, such as *Rhizoctonia solani*, *Fusarium solani*, *Pythium aphanidermatum*, and *Pythium ultimum* [25,26,27,28]. Any of these pathogens can cause root rot disease in tomato, leading to huge economic and yield losses. Tomato root rot caused by *Rhizoctonia solani*, also known as Rhizoctonia root rot, is a major limiter of the growth and production of tomato. The growth parameters of tomato plants, such as shoot and root fresh weights, shoot and root length, and yield, are influenced after infection by *R. solani*. Solanki et al. reported that the total yield of tomato (healthy control of 116.04 q ha^−1^) was significantly reduced by *R. solani* infection (38.3 q ha^−1^) under field conditions [29]. Similarly, in another study by Solanki et al., the yield of tomato was dramatically reduced by *R. solani* infection (28.89 q ha^−1^) compared to that of healthy plants (168.89 q ha^−1^) [30]. Thus, this is highlighted and discussed in this review.

The pathogen *R. solani* belongs to the phylum Basidiomycota, family Ceratobasidiaceae, and genus *Rhizoctonia* [31]. *R. solani* species can be classified into different anastomosis groups (AGs); different AGs of *R. solani* vary in their morphological characteristics and pathogenic virulence [32,33]. *R. solani* has a wide range of hosts and can infect the plant families of Linaceae, Araceae, Poaceae, Fabaceae, Malvaceae, Solanaceae, Amaranthaceae, Rubiaceae, Brassicaceae, Asteraceae, and Moraceae, affecting nearly 250 plant species [34,35,36]. *R. solani* can cause root rot, crown rot, seed rot, stem rot, pod rot, limb rot, hypocotyl rot, head rot, bottom rot, pre-emergence and post-emergence damping off, black scurf, blights, and cankers in different host plant species [34,35,36]. *R. solani* can also exhibit strong competition with other saprophytes [34].

A notable property of *R. solani* is that it can produce sclerotia, which are formed from undifferentiated hyphae or monilioid cells [36]. Sclerotia are formed in three development stages: the initiation, development, and maturation stages [36]. The sclerotia of *R. solani* can resist harsh environmental conditions and persist in soil or plant debris for many years. Under appropriate conditions, such as warm and high-humidity environments, sclerotia can germinate into mycelia and infect the plant roots, thereby leading to disease symptoms and even causing plant death [34]. Sclerotia can form again on the infected plant debris. Sclerotia in the soil and plant debris can act as an inoculum source for the further infection of host plants [34].

Different control strategies are applied to manage *Rhizoctonia* root rot, with the commonly used methods being chemical and biological control (biocontrol). Chemical fungicides are commonly used to control plant diseases because they offer rapid efficacy and a wide control spectrum; however, they pose a threat to the environment, can induce pathogen resistance, and can affect food safety, seriously limiting their application [37,38,39]. Compared with chemical control, biocontrol has the advantages of being green, environmentally friendly, safe, and suitable for sustainable application; thus, it has drawn much attention [40,41,42]. Biocontrol usually refers to the management of plant diseases through the application of useful microorganisms, including bacteria and fungi. Bacteria such as *Bacillus* species, *Pseudomonas fluorescens*, and *Streptomyces* species and fungi such as *Trichoderma* species, *Clonostachys rosea*, and *Coniothyrium minitans* are commonly used as biocontrol agents to manage plant diseases [43,44,45,46,47,48]. The biocontrol mechanism involves the secretion of cell-wall-degrading enzymes, the production of metabolites, the induction of plant systemic resistance, and competition for nutrients and space [49,50,51,52]. To date, several biocontrol microorganisms comprising bacteria (e.g., *Bacillus*, *Pseudomonas*, *Streptomyces*) and fungi (e.g., *Trichoderma*, *Paecilomyces*) have been reported to be effective in controlling tomato root rot caused by *R. solani* [30,53,54,55]. Although there are many reviews about *R. solani* and tomatoes, comprehensive reviews about the biocontrol of Rhizoctonia root rot in tomato using biocontrol microorganisms, along with their biocontrol mechanisms, remain scarce.

“Tomato root rot” and “*Rhizoctonia solani*” were used as keywords to search the Web of Science and PubMed databases for all types of studies for this review. Studies about the biological characters, pathogenicity, and damage caused by *R. solani* were included, as well as all studies related to the control of tomato root rot caused by *R. solani* using biocontrol microorganisms across various experimental scales such as in plate, greenhouse, and field conditions.

This review provides a comprehensive summary and analysis of the management of tomato root rot caused by *R. solani* through the use of biocontrol methods. Bacterial and fungal biocontrol agents that can control tomato Rhizoctonia root rot are discussed in detail, and their potential biocontrol mechanisms are explored. Finally, future perspectives on the biocontrol of tomato Rhizoctonia root rot are presented. This review provides useful information for the biocontrol of tomato root rot caused by *R. solani*.

## 2. Biocontrol Bacteria in Management of Tomato Root Rot Caused by *R. solani*

Several bacterial agents, such as *Bacillus*, *Pseudomonas*, *Streptomyces*, *Enterobacter*, *Alcaligenes*, *Paenebacillus*, and *Acinetobacter*, have been used to control tomato root rot caused by *R. solani* (Table 1).

Numerous *Bacillus* species, including *B. subtillis*, *B. thuriengiensis*, *B. megaterium*, *B. amyloliquefaciens*, and *B. velezensis*, have exhibited excellent efficacy in controlling Rhizoctonia root rot in tomato, as well as in promoting tomato growth. Rashad et al. found that *B. subtilis* SR22, isolated from faba bean stem, could significantly reduce the severity of root rot in *R. solani*-infected tomato plants compared with that in *R. solani*-infected controls. Moreover, growth parameters, namely, shoot length, root length, shoot dry weight, and root dry weight, remarkably increased after the application of SR22 in Rhizoctonia root-rot-infected tomato plants [56]. *B. subtilis* strains MB14 and MB99 isolated from tomato rhizosphere soil, which can produce the cell-wall-degrading enzymes chitinase, β-1,3-glucanase, β-1,4-glucanase, and protease, exhibited excellent effects in suppressing tomato root rot caused by *R. solani* under greenhouse conditions. The field-scale condition showed that MB14 could significantly reduce the disease index of root rot in tomatoes, as well as increase the number of fruits/plants and yield, compared with *R. solani*-infected controls [30]. Moreover, another study found that four genes related to antibiotic production in MB14 were amplified and exhibited similarity to surfactin synthetase, ilutrin A synthetase, fengycin synthetase, and mycosubtilin synthase subunit B, which might be an important mechanism of MB14 in suppressing tomato root rot caused by *R. solani* [57].

*B. subtilis* B10 significantly reduced the disease index of root rot caused by *R. solani* in two tomato cultivars, namely, Marmande and Rio Grande, over two years compared with a fungicide. B10 also improved the growth parameters of the two tomato cultivars, namely, plant height, aerial part fresh weight, and root growth [58]. El-Gamal et al. reported that a *B. subtilis* strain isolated from biocompost rice straw significantly suppressed the disease incidence of root rot in *R. solani*-infected tomatoes compared with *R. solani*-infected controls under greenhouse conditions [59].

In addition to *B. subtilis*, other *Bacillus* species have also been found to suppress tomato root rot caused by *R. solani*. The application of *B. velezensis* strains significantly reduced the incidence of tomato Rhizoctonia root rot. Moreover, tomato plants treated with *B. velezensis* showed increases in growth parameters, namely, root and shoot fresh weight, root dry weight, and the number of fruits, compared with *R. solani*-infected controls [60]. Two endophytic bacteria, *B. velezensis* L1 and *B. megaterium* L2, isolated from strawberry plants, effectively controlled tomato root rot disease caused by *R. solani* in pots maintained in greenhouse conditions [61]. A *B. megaterium* strain, MB3, also effectively controlled tomato root rot caused by *R. solani* under greenhouse conditions. *B. amyloliquefaciens* MB101 significantly reduced the disease index of tomato root rot caused by *R. solani* under both greenhouse and field conditions [30]. *B. thuringiensis* B2 also exhibited a dual effect on two tomato cultivars over two years by suppressing the disease index of Rhizoctonia root rot and promoting tomato growth parameters [58].

*P. fluorescens* and *P. aeruginosa* are the two main *Pseudomonas* species that suppress the incidence of tomato root rot caused by *R. solani*. In addition, some *Pseudomonas* species promote tomato growth. The application of *P. fluorescens* A6RI to *R. solani*-infected tomato plants significantly reduced both the external hyphae appressed to epidermal cells and the intraradical infection caused by *R. solani* on tomato roots. Tomatoes treated with A6RI also exhibited remarkably increased growth parameters, including fresh root and shoot weights, and root morphogenetic parameters, including total root length, root branching degree, and the number of root tips, compared with *R. solani*-infected controls [62]. *P. fluorescens* MPF47 showed high growth inhibition against *R. solani*, and it significantly reduced the disease index of tomato root rot caused by *R. solani* compared with infected controls under glasshouse conditions. Moreover, MPF47 also increased the root and shoot lengths, total fruit numbers, and yield of tomato compared with *R. solani*-infected controls [29]. Other *P. fluorescens* strains in different reports also exhibited excellent effects in reducing the incidence of tomato root rot caused by *R. solani* [59,63].

*P. aeruginosa* MPF14 and MB123, isolated from tomato rhizosphere soil, can inhibit the growth of *R. solani*. An experiment conducted under glasshouse conditions showed that the disease index of Rhizoctonia root rot was significantly reduced in tomatoes treated with MPF14 and MB123 compared with *R. solani*-infected controls. A further study revealed that both strains could produce cell-wall-degrading enzymes, including chitinase, β-1,3-glucanase, and protease. MPF14 and MB123 also increased tomato growth parameters, namely, root and shoot lengths, total fruit numbers, and yield [29]. The application of *P. aeruginosa* IE-6 could reduce the infection of tomato root rot caused by *R. solani*, as well as promote tomato growth in terms of plant height and shoot weight [55]. Similarly, *P. aeruginosa* PGPR11 could also control tomato root rot caused by *R. solani* and promote tomato plant growth [64]. A *Pseudomonas* strain EFP-47 isolated from *Digera muricata* root showed a promising effect in suppressing tomato root rot caused by *R. solani* [53,65]. Moreover, *Pseudomonas* strains of MRFP-205, MRFP-206, MRFP-212 and EFP-171 were reported having the ability to reduce the infection of *R. solani* in tomato plant [53,66]. Solanki et al. reported two strains producing cell-wall-degrading enzymes, *Pseudomonas* sp. M10A and MB65, which showed dual effects of suppressing tomato Rhizoctonia root rot and promoting plant growth [29].

In addition to *Bacillus* and *Pseudomonas*, *Streptomyces* is also an important biocontrol agent in the control of plant diseases [67,68,69,70,71]. *S. atrovirens* N23 significantly reduced the disease index of tomato root rot caused by *R. solani* under field conditions. Moreover, the application of N23 notably increased the plant growth parameters of *R. solani*-infected tomato plants, namely, root length, plant height, plant biomass, fruits per plant, and fruit biomass per plant. Further study revealed that the activities of plant-defense-related enzymes, including chitinase, β-1,3-glucanase, phenylalanine ammonia lyase, polyphenol oxidase, and peroxidase, increased in *R. solani*-infected tomato plants treated with N23 compared with *R. solani*-infected controls [54]. A *Streptomyces* species, *S. vinaceusdrappus* S5MW2, which was isolated from Chilika lake and could produce chitinase, inhibited the growth of *R. solani* in a dual-culture plate, and it showed excellent effects in suppressing tomato root rot caused by *R. solani* under greenhouse conditions. Tomatoes treated with S5MW2 also exhibited improvements in growth parameters, namely, root and shoot lengths and plant fresh weight, compared with *R. solani*-infected controls. SEM observation showed that the *R. solani* mycelia had been destructed by the application of S5MW2 [72].

Other bacterial agents, namely, *Enterobacter cloacae* B16, *Alcaligenes faecalis* MUN1 and MB21, *Alcaligenes* sp. MPF37, *Enterobacter* sp. MPM1 isolated from tomato rhizosphere soils, and *Herpaspirillum huttiense* S7 isolated from strawberry plants, as well as *Bradyrhizobium japonicum* KUCC 569 and *Acinetobacter pittii*, exhibited effects in reducing the infection of tomato root rot caused by *R. solani*. Many of these strains also promoted the growth of tomato plants [29,58,60,61,64]. In addition, the application of S7 significantly increased the activities of antioxidant enzymes [61]. Similarly, the activities of antioxidant defense enzymes in *R. solani*-infected tomato plants also increased after the application of *Acinetobacter pittii* [60].

Although experimental factors such as the inoculum concentrations of biocontrol microorganisms and *R. solani*, soil types, experimental scale, and environmental conditions vary, along with diverse disease evaluation indicators, the reported microorganisms effectively control tomato root rot caused by *R. solani*.

The biocontrol ability of several *Bacillus* strains, *Pseudomonas* strains, and a *Streptomyces* *atrovirens* strain against tomato root rot caused by *R. solani* was evaluated under field conditions. Among these biocontrol bacteria, *P. aeruginosa* IE-6 exhibited an excellent control ability, being able to completely inhibit tomato root rot caused by *R. solani* at 30 and 60 d [55]. In addition, *B. amyloliquefaciens* MB101, *Pseudomonas* sp. MRFP-205, and EFP-47, as well as *Streptomyces atrovirens* N23, strongly suppressed tomato root rot caused by *R. solani*. Compared with an *R. solani* infection control, the disease index was reduced from 65.83% to 29.17% by using MB101 and reduced from 68.23% to 33.00% by using N23 [30,54]. The infection value was reduced from 31.2% to 6.2% with the application of both MRFP-205 and EFP-47 [53].

More *Bacillus* and *Pseudomonas* strains exhibited control capacities in suppressing tomato root rot caused by *R. solani* under greenhouse/glasshouse/screen house scales. The disease severity was reduced from 65.0% (*R. solani* infection control) to 30.0% by using *B. subtilis* SR22 [56]. Similarly, *B. velezensis* L1 and *B. megaterium* L2 could reduce the disease index from 85% (*R. solani* infection control) to 22.5% and 27.5%, respectively [61]. *P. aeruginosa* PGPR11 could completely prevent the infection of tomato by *R. solani* [64]. *P. aeruginosa* MB123 and *P. fluorescens* MPF47 also exhibited high control effects against tomato root rot caused by *R. solani*, with the disease index reduced from 71.54% (*R. solani* infection control) to 28.31% and 18.62% after the application of MB123 and MPF47, respectively [29]. Besides *Bacillus* and *Pseudomonas*, *Bradyrhizobium japonicum* KUCC 569 and *Herpaspirillum huttiense* S7 could effectively suppress tomato root rot caused by *R. solani*. KUCC 569 could completely prevent *R. solani*’s infection of tomato plants, and S7 could reduce the disease index from 85% (*R. solani* infection control) to 25% [61,64].

**Table 1:** Overview of biocontrol bacterial agents showing control ability against tomato root rot.

Biocontrol Bacteria	Strain Name	Isolation Source	Application Scale	^1^ Disease Indicators (%)	^2^ Disease Indicators (%)
***Bacillus***					
*B. subtillis*	MB14 [30]	Tomato rhizospheric soil	Field	65.83 (Disease index)	37.50 (Disease index)
*B. amyloliquefaciens*	MB101 [30]	Tomato rhizospheric soil	Field	65.83 (Disease index)	29.17 (Disease index)
*B. thuriengiensis*	B2 [58]	Tomato rhizospheric soil	Greenhouse	100 (Disease incidence)	80 (Disease incidence)
*B. subtillis*	B10 [58]	Tomato rhizospheric soil	Greenhouse	100 (Disease incidence)	66.66 (Disease incidence)
*B. subtilis*	SR22 [56]	Faba bean stem	Greenhouse	65.0 (Disease severity)	30.0 (Disease severity)
*B. subtillis*	MB99 [30]	Tomato rhizospheric soil	Greenhouse	71.94 (Disease index)	48.89 (Disease index)
*B. subtilis*	- [59]	Biocompost rice straw	Greenhouse	52.0 (Disease incidence)	24.0 (Disease incidence)
*B. megaterium*	MB3 [30]	Tomato rhizospheric soil	Greenhouse	71.94 (Disease index)	41.67 (Disease index)
*B. velezensis*	L1 [61]	strawberry	Greenhouse	85 (Disease index)	22.5 (Disease index)
*B. megaterium*	L2 [61]	strawberry	Greenhouse	85 (Disease index)	27.5 (Disease index)
***Pseudomonas***					
*P. aeruginosa*	IE-6 [55]	Sunflower root	Field	58 (Infection)	0 (Infection)
*Pseudomonas* sp.	MRFP-205 [53]	-	Field	31.2 (Infection)	6.2 (Infection)
*Pseudomonas* sp.	MRFP-206 [53]	-	Field	31.2 (Infection)	18.7 (Infection)
*Pseudomonas* sp.	MRFP-212 [53]	-	Field	31.2 (Infection)	18.7 (Infection)
*Pseudomonas* sp.	EFP-47 [53]	*Digera muricata* root	Field	31.2 (Infection)	6.2 (Infection)
*P. fluorescens*	- [63]	-	Greenhouse	86.7 (Disease severity)	50.7 (Disease severity)
*Pseudomonas* sp.	M10A [29]	Tomato rhizosphere soil	Glasshouse	71.54 (Disease index)	43.53 (Disease index)
*Pseudomonas* sp.	MB65 [29]	Tomato rhizosphere soil	Glasshouse	71.54 (Disease index)	46.03 (Disease index)
*P. aeruginosa*	MB123 [29]	Tomato rhizosphere soil	Glasshouse	71.54 (Disease index)	28.31 (Disease index)
*P. aeruginosa*	MPF14 [29]	Tomato rhizosphere soil	Glasshouse	71.54 (Disease index)	48.18 (Disease index)
*P. fluorescens*	MPF47 [29]	Tomato rhizosphere soil	Glasshouse	71.54 (Disease index)	18.62 (Disease index)
*P. fluorescens*	- [59]	Biocompost rice straw	Greenhouse	52.0 (Disease incidence)	22.0 (Disease incidence)
*Pseudomonas* sp.	EFP-171 [66]	-	Screen house	31.2 (Infection)	18.7 (Infection)
*P. aeruginosa*	PGPR11 [64]	*Ficus carica* L. roots	Screen house	37.5 (Infection)	0 (Infection)
***Enterobacter***					
*E. cloacae*	B16 [58]	Tomato rhizospheric soil	Greenhouse	100 (Disease incidence)	53.33 (Disease incidence)
*Enterobacter* sp.	MPM1 [29]	Tomato rhizosphere soil	Glasshouse	71.54 (Disease index)	50.32 (Disease index)
***Alcaligenes***					
*A. faecalis*	MB21 [29]	Tomato rhizosphere soil	Glasshouse	71.54 (Disease index)	48.69 (Disease index)
*A. faecalis*	MUN1 [29]	Tomato rhizosphere soil	Glasshouse	71.54 (Disease index)	43.53 (Disease index)
*Alcaligenes* sp.	MPF37 [29]	Tomato rhizosphere soil	Glasshouse	71.54 (Disease index)	48.18 (Disease index)
**Other Microorganisms**					
*Streptomyces atrovirens*	N23 [54]	-	Field	68.23 (Disease index)	33.00 (Disease index)
*Herpaspirillum huttiense*	S7 [61]	strawberry	Greenhouse	85 (Disease index)	25 (Disease index)
*Bradyrhizobium japonicum*	KUCC 569 [64]	-	Screen house	37.5 (Infection)	0 (Infection)

Note: “-” represents not available. ^1^ Disease indicators (%) and ^2^ Disease indicators (%) mean disease indicators in CK group (infected) and treatment group (application of biocontrol agents). Biocontrol bacteria with disease indicator values were listed.

## 3. Biocontrol Fungi in Management of Tomato Root Rot Caused by *R. solani*

Fungal biocontrol agents such as *Trichoderma*, *Paecilomyces*, *Penicillium*, *Pythium*, and *Muscodor* have been found to decrease the incidence of tomato root rot caused by *R. solani* (Table 2). Among these, *Trichoderma* strains are the most frequently reported with regard to the control of tomato *Rhizoctonia* root rot.

Three *T. harzianum* strains, T1, T2, and T4, isolated from the rhizosphere, together with another *T. harzianum* strain, T-22, exhibited significant effects in reducing the incidence of tomato root rot caused by *R. solani* [73]. Two *T. harzianum* strains, ET-6 isolated from *Lantana camara* leaf and ET-9 isolated from *Leucas aspera* root, reduced the infection of tomato root rot caused by *R. solani* under field conditions at 45 days of treatment in two consecutive years [53].

An endophytic strain, *T. viride* ET-4, isolated from *Euphorbia hirta* root, exhibited a dual effect on tomato plants by reducing the infection of root rot caused by *R. solani* and increasing the shoot and root lengths and weights [66]. A *T. viride* strain had a dual effect of disease control and plant growth promotion. *T. viride* effectively inhibited the mycelial growth of *R. solani* and remarkably reduced the severity of tomato root rot caused by *R. solani* under greenhouse conditions. Additionally, the *T. viride* strain had a positive effect on tomato growth by increasing the root and shoot dry weights. Further study revealed that the chitinase activity induced by *T. viride* against *R. solani* was significantly higher than in the control, which might be an important mechanism underlying the effects of *T. viride* against *R. solani* [63]. In addition, six biocontrol agents, namely, *T. hamatum* (NAIMCC-F-01721), *H. lixii* (NAIMCC-F-01735), *H. lixii* (NAIMCC-F-01760), *T. asperellum* (NAIMCC-F-01763), *H. virens* (NAIMCC-F-01775), and *H. lixii* (NAIMCC-F-01809), capable of producing cell-wall-degrading enzymes (chitinase, β-1,3 glucanase, and protease), exhibited effects in decreasing the index of tomato root rot caused by *R. solani* under greenhouse conditions. Moreover, the six biocontrol agents promoted tomato growth in terms of the length and dry weight of both the root and shoot. Furthermore, the activities of defense enzymes, including peroxidase, polyphenol oxidase, and phenylalanine ammonia lyase, also increased in *R. solani*-infected tomato plants treated with the six biocontrol agents [74]. Meanwhile, some of the above strains could also exhibit control ability in salt stress conditions under greenhouse conditions [75].

In addition to the abovementioned strains, *T. pubescens*, *T. asperelloides*, and *T. polysporum* also exhibited effects in controlling tomato root rot caused by *R. solani*. *T. pubescens* Tp21, isolated from rhizospheric soil, exhibited inhibition effects on *R. solani* in vitro and significantly reduced the disease index of tomato root rot caused by *R. solani* in vivo compared with a control. Further study revealed that the application of Tp21 also notably increased the activities of superoxide dismutase, polyphenol oxidase, peroxidase, and catalase. HPLC analysis found that the application of Tp21 increased the contents of polyphenolic compounds of tomato [76]. *T. asperelloides* Ta41 also significantly reduced the disease index of tomato root rot caused by *R. solani* compared with the infection control. A microscopic observation found that penetration structures, such as penetration peg, formed in the Ta41 hyperparasite of *R. solani*, and then Ta41 coiled around the cell wall of *R. solani* and penetrated *R. solani* hyphae. Further study revealed that the expression levels of defense-related genes, namely PR-1, PR-2, PR-3, and CHS, encoding pathogenesis-related protein-1, endoglucanase, chitinase, and chalcone synthase, respectively, remarkably improved after the application of Ta41 compared with the control [77]. An endophytic strain, *T. polysporum* ET-19, isolated from *Tradescantia pallida* stem, reduced the infection of tomato root rot caused by *R. solani* [66].

*Paecilomyces formosus* ED isolated from tomato root exhibited high biocontrol efficacy in suppressing tomato root rot caused by *R. solani*. The activities of antioxidant enzymes such as peroxidase and polyphenol oxidase improved after the application of high concentrations of ED spores. Histopathological analysis found that the cell wall of the tomato was broken after *R. solani* infection and that it was restored after applying ED. Moreover, the plant height, total number of leaves/plants, and biomass (wet weight) of tomato were significantly increased after the application of ED at high inoculum concentrations [78]. Siddiqui et al. reported that a *Paecilomyces* species, *P. lilacinus* PI, had a notable effect on suppressing tomato root infection caused by *R. solani* at 60 days [55].

Other biocontrol fungi, such as *Pythium oligandrum*, *Glomus mosseae*, *Acrophialophora jodhpurensis*, *Muscodor cinnamomic*, and *Penicillium*, exhibited excellent effects in controlling tomato root rot disease caused by *R. solani*. *P. oligandrum* Po37 was isolated from the grapevine rhizosphere and exhibited effects in reducing the incidence of Rhizoctonia root rot in two tomato cultivars. In addition to suppressing Rhizoctonia root rot, Po37 also improved tomato plant growth parameters, including root fresh weight, plant height, and aerial part fresh weight [79]. The application of *Glomus mosseae* BEG12 notably affected the rate of *R. solani* infecting tomato roots by reducing the intraradical infection and the external hyphae appressed to epidermal cells by *R. solani*. Moreover, the fresh root and shoot weights, together with the total root length and number of root tips, significantly increased in Rhizoctonia root rot tomato plants treated with BEG12 compared with *R. solani*-infected controls [62]. *Acrophialophora jodhpurensis* significantly reduced the disease index of tomato plants infected by *R. solani*. A light microscopic observation found deformation of the hyphae and cytoplasm lysis of *R. solani* mycelia after the application of *A. jodhpurensis*. SEM showed that the mycelia of *R. solani* were dense with a rough surface and severely distorted after using *A. jodhpurensis*. TEM showed that, compared with no *A. jodhpurensis* treatment, the hyphal cells of *R. solani* were clearly altered after using *A. jodhpurensis*. The activities of antioxidant enzymes, including guaiacol peroxidase, catalase, and ascorbate peroxidase, were higher in tomato plants treated with *A. jodhpurensis* than in *R. solani*-infected controls [80]. The *Penicillium* strain Pen1-R, isolated from potato, exhibited a dual effect on tomato plants by reducing the root rot infected by *R. solani* under field conditions and promoting growth parameters [53]. The endophytic fungus *Muscodor cinnamomi* CMU-Cib461, obtained from *Cinnamomum bejolghota* (Buch.-Ham.) Sweet, completely suppressed the incidence of tomato root rot caused by *R. solani*. Moreover, CMU-Cib461 could produce indole-3-acetic acid and increase the shoot dry weight, root dry weight, and root length of tomato plants [81]. Eight fungi, including *T. viride*, *T. harzianum*, *T. hamatum*, *Aspergillus versicolar*, *A. terreus*, *Talaromyces wortmanni*, *Chaetomium* spp., and *Epicoccum* sp., exhibited antagonistic effects on the growth of *R. solani* [82].

Additionally, three endophytic yeast strains—*Debaryomyces hansenii* Y-17 and Y-34 isolated from *Azadirachta indica*, and *Meyerozyma guilliermondii* Y-62 isolated from *Carica papaya*—could reduce the infection of tomato plants caused by *R. solani* at both 45 and 90 d in a field plot experiment. All three yeast strains could improve the vegetative growth of tomato plants in terms of shoot and root length and shoot and root weight. The application of the three yeast strains could also improve the antioxidant activity and polyphenols compared with the control [83].

*Trichoderma* strains, together with three yeast strains and *Penicillium* sp. Pen1-R, exhibited excellent control effects on suppressing tomato root rot caused by *R. solani* under field conditions. Compared with *R. solani* infection control, *T. harzianum* ET-6 could reduce the infection value from 31.2% to 6.2%, and *T. lixii* NAIMCC-F-01760 could reduce the disease index from 68.23% to 28% [53,54]. Three yeast strains, *Debaryomyces hansenii* Y-17, Y-34 and *Meyerozyma guilliermondii* Y-62, could reduce the infection value from 87.5% (*R. solani* infection control) to 37.5%, 37.5%, and 25%, respectively [83]. Similarly, *Penicillium* sp. Pen1-R could also reduce the infection value from 31.2% (*R. solani* infection control) to 6.2% under the field condition [53].

*Trichoderma* strains are the most reported strains that can effectively inhibit tomato root rot caused by *R. solani* under greenhouse/climate room conditions. Four *T. harzianum* strains, T1, T2, T4, and T-22, could reduce the disease incidence from 50% (*R. solani* infection control) to 18.7%, 15.6%, 23.4%, and 17.2%, respectively [73]. Compared with the *R. solani* infection control, the disease index was reduced from 72.78% to 33.33% and 23.33% through the application of *T. hamatum* NAIMCC-F-01721 and *T. asperellum* NAIMCC-F-01763, respectively [74]. Similarly, the disease index was reduced from 78.67% to 16% by using *T. pubescens* Tp21, and reduced from 81% to 16% by using *T. asperelloides* Ta41 [76,77]. Moreover, three *Hypocrea* strains, *H. lixii* NAIMCC-F-01735, NAIMCC-F-01809, and *H. virens* NAIMCC-F-01775, could reduce the disease index from 72.78% (*R. solani* infection control) to 31.12%, 28.89%, and 26.11%, respectively [74].

**Table 2:** Overview of biocontrol fungal agents showing control ability against tomato root rot.

Biocontrol Fungi	Strain Name	Isolation Source	Application Scale	^1^ Disease Indicators (%)	^2^ Disease Indicators (%)
***Trichoderma***					
*T. harzianum*	ET-6 [53]	*Lantana camara* leaf	Field	31.2 (Infection)	6.2 (Infection)
*T. harzianum*	ET-9 [53]	*Leucas aspera* root	Field	31.2 (Infection)	18.7 (Infection)
*T. harzianum*	T1 [73]	Rhizosphere	Climate room	50 (Disease incidence)	18.7 (Disease incidence)
*T. harzianum*	T2 [73]	Rhizosphere	Climate room	50 (Disease incidence)	15.6 (Disease incidence)
*T. harzianum*	T4 [73]	Rhizosphere	Climate room	50 (Disease incidence)	23.4 (Disease incidence)
*T. harzianum*	T-22 [73]	-	Climate room	50 (Disease incidence)	17.2 (Disease incidence)
*T. lixii*	NAIMCC-F-01760 [54]	-	Field	68.23 (Disease index)	28 (Disease index)
*T. hamatum*	NAIMCC-F-01721 [74]	-	Greenhouse	72.78 (Disease index)	33.33 (Disease index)
*T. asperellum*	NAIMCC-F-01763 [74]	-	Greenhouse	72.78 (Disease index)	23.33 (Disease index)
*T. pubescens*	Tp21 [76]	Rhizospheric soil	Greenhouse	78.67 (Disease index)	16 (Disease index)
*T. asperelloides*	Ta41 [77]	Rhizosphere soil	Greenhouse	81 (Disease index)	16 (Disease index)
*T. viride*	- [63]	-	Greenhouse	86.7 (Disease severity)	58.3 (Disease severity)
*T. viride*	ET-4 [66]	*Euphorbia hirta* root	Screen house	31.2 (Infection)	25 (Infection)
*T. polysporum*	ET-19 [66]	*Tradescantia pallida* stem	Screen house	31.2 (Infection)	25 (Infection)
***Hypocrea***					
*H. lixii*	NAIMCC-F-01735 [74]	-	Greenhouse	72.78 (Disease index)	31.12 (Disease index)
*H. lixii*	NAIMCC-F-01809 [74]	-	Greenhouse	72.78 (Disease index)	28.89 (Disease index)
*H. virens*	NAIMCC-F-01775 [74]	-	Greenhouse	72.78 (Disease index)	26.11 (Disease index)
***Debaryomyces***					
*Debaryomyces hansenii*	Y-17 [83]	*Azadirachta indica*	Field plot	87.5 (Infection)	37.5 (Infection)
*D. hansenii*	Y-34 [83]	*Azadirachta indica*	Field plot	87.5 (Infection)	37.5 (Infection)
**Other Microorganisms**					
*Penicillium* sp.	Pen1-R [53]	Potato	Field	31.2 (Infection)	6.2 (Infection)
*Paecilomyces lilacinus*	PI [55]	-	Field	58 (Infection)	33 (Infection)
*Meyerozyma guilliermondii*	Y-62 [83]	*Carica papaya*	Field plot	87.5 (Infection)	25 (Infection)
*Pythium oligandrum*	Po37 [79]	Grapevine rhizosphere	Greenhouse	100 (Disease incidence)	67 (Disease incidence)

Note: “-” represents not available. ^1^ Disease indicators (%) and ^2^ Disease indicators (%) mean disease indicators in CK group (infected) and treatment group (application of biocontrol agents). Biocontrol fungi with disease indicator values were listed.

## 4. Biocontrol Mechanisms of Controlling Tomato Root Rot Caused by *R. solani*

**Figure 1:**
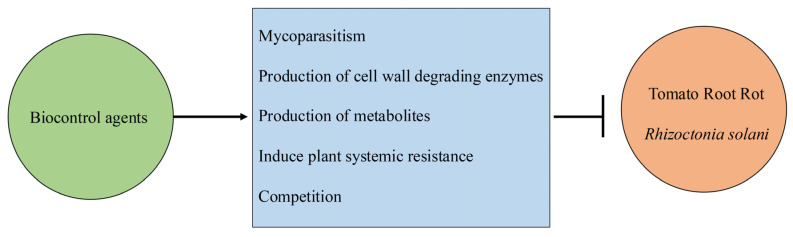
Mechanisms of biocontrol agents against tomato root rot caused by *Rhizoctonia solani.*

Understanding the biocontrol mechanisms involved is helpful for further enhancing the control efficacy of biocontrol agents against tomato root rot caused by *R. solani*. Biocontrol mechanisms commonly involve the secretion of cell-wall-degrading enzymes, the production of metabolites, mycoparasitism, the induction of plant systemic resistance, and competition (Figure 1).

### 4.1. Secretion of Cell-Wall-Degrading Enzymes

Cell-wall-degrading enzymes secreted by biocontrol agents can damage the cell wall of *R. solani*, thereby playing important roles in the control of tomato Rhizoctonia root rot. Nine bacteria, namely, *Pseudomonas* sp. M10A and MB65, *P. aeruginosa* MPF14 and MB123, *P. fluorescens* MPF47, *Alcaligenes faecalis* MUN1 and MB21, *Alcaligenes* sp. MPF37, and *Enterobacter* sp. MPM1, could produce the cell-wall-degrading enzymes chitinases, β-1,3-glucanases, and proteases and exhibited excellent control effects on tomato root rot [29]. *Paecilomyces formosus* ED, which can suppress tomato root rot, could produce chitinase, cellulase, and protease [78]. El-Gamal et al. found that both *B. subtilis* and *P. fluorescens* strains could produce β-1,3 glucanase, β-1,4 glucanase, and chitinase, which might play essential roles in their antagonistic activity [59].

### 4.2. Production of Metabolites

Biocontrol agents can also control tomato root rot by producing various metabolites. Rashad et al. found that metabolites with an antifungal background, such as chlorogenic acid, pyrrolo [1,2-a]pyrazine-1,4-dione, propyl thioglycolic acid, and phthalic acid, were produced by *B. subtilis* SR22 and might be associated with its antagonistic ability [56]. Daroodi et al. found that *Acrophialophora jodhpurensis* could produce volatile and non-volatile metabolites that inhibit the mycelial growth of *R. solani* [80].

### 4.3. Mycoparasitism

Mycoparasitism is another important mechanism of biocontrol agents in the management of tomato root rot. A microscopic observation found that *P. oligandrum* Po37 coiled around the hyphae of *R. solani* and that the infection pegs formed and penetrated *R. solani* cells [79]. An SEM observation showed that *H. lixii* NAIMCC-F-01760 coiled and deformed the mycelia of *R. solani* [84].

### 4.4. Induction of Plant Systemic Resistance

The induction of plant systemic resistance is an important mechanism by which biocontrol agents inhibit root rot in tomato plants. The expression levels of several defense-related genes and the activities of antioxidant enzymes were increased after applying biocontrol agents against tomato root rot, which might be associated with the activation of plant systemic resistance. Behiry et al. found that the application of *T. pubescens* Tp21 to tomato could significantly reduce the disease index of root rot disease caused by *R. solani*. Meanwhile, the expression levels of the defense-related genes *PAL*, *CHS*, and *HQT*, which encode phenylalanine ammonia-lyase, chalcone synthase, and hydroxycinnamoyl Co A quinate hydroxycinnamoyl transferase, respectively, as well as the activities of the antioxidant enzymes polyphenol oxidase, peroxidase, catalase, and superoxide dismutase, were significantly improved when using Tp21 against *R. solani* compared with *R. solani* infection only [76]. Similarly, Rashad et al. found that the disease incidence of tomato root rot caused by *R. solani* was dramatically reduced by using *B. subtilis* SR22. Deep study found that the expression levels of the defense response genes *JERF3* (encoding jasmonate and ethylene-responsive factor 3), *POD* (encoding peroxidase), and *PR1* (encoding pathogenesis-related protein 1) and the activities of the antioxidant enzymes polyphenol oxidase and peroxidase notably increased after the application of *B. subtilis* SR22 to tomato roots infected by *R. solani* [56].

### 4.5. Competition

Competition for nutrients or space is also an important mechanism by which biocontrol microorganisms act against plant pathogens. Solanki et al. investigated the role of the nutrient competition of three biocontrol agents *B. amyloliquefaciens* MB101, *S. atrovirens* N23 and *T. lixii* NAIMCC-F-01760 against the tomato root rot pathogen *R. solani*, and found that nutrient supplementation could influence the antagonistic ability of the biocontrol microorganisms; moreover, nutrient competition could also improve the production of cell-wall-degrading enzymes [84].

## 5. Conclusions and Prospects

Tomato Rhizoctonia root rot is a soilborne disease caused by *R. solani*; it can seriously affect tomato production and lead to huge economic losses. Biocontrol has received considerable attention because it is green, safe, and sustainable. This review mainly discusses the biocontrol agents that can control tomato root rot caused by *R. solani*, including bacterial agents, such as *Bacillus*, *Pseudomonas*, *Streptomyces*, *Enterobacter*, *Alcaligenes*, *Paenebacillus*, and *Acinetobacter*, and fungal agents, such as *Trichoderma*, *Paecilomyces*, *Penicillium*, *Pythium*, and *Muscodor*, as well as their potential biocontrol mechanisms. This review provides a basis for further field application of biocontrol agents to suppress tomato root rot caused by *R. solani*.

The most important method for controlling tomato root rot caused by *R. solani* is the practical application of biocontrol microorganisms, which necessitates the consideration of their formulation, reproducibility under field conditions, environmental variability, regulatory considerations for some microbial agents, and limitations. Different formulations of biocontrol agents are important for applications in various field conditions, as well as agent stability and shelf life. The commonly used microbial formulations are liquid formulations, powder formulations, and granule formulations, etc., with liquid formulations being the most reported for the management of tomato root rot caused by *R. solani* [29,54].

Reproducibility under field conditions is also a crucial factor for the practical application and commercialization of biocontrol microorganisms. Reproducibility could ensure the stable and reliable effects of biocontrol agents against plant pathogens, as factors such as weather, season, soil type, and other environmental parameters vary. Zehra et al. investigated the ability of several biocontrol microorganisms to manage the infection of tomato plants by *R. solani* under field conditions over two years. The results showed that *Pseudomonas* MRFP-205, MRFP-206, MRFP-212, EFP-47, *Penicillium* Pen1-R, *Trichoderma* ET-6 and ET-9 exhibited effective control ability in two years [53]. In addition, regulatory considerations for some microbial agents are necessary for practical application of biocontrol agents, mainly including safety, environmental risk, and manufacturing quality.

Some limitations exist in the current application of biocontrol microorganisms against tomato root rot caused by *R. solani*. Field assays are necessary to verify the control effects of biocontrol agents and for their further commercialization; however, the control efficacy of most current biocontrol agents that are capable of controlling tomato root rot caused by *R. solani* is investigated under greenhouse or plate antagonism conditions. In addition, commercial microbial products are rare, and the formulations of microbial agents are insufficient. Li et al. investigated the control effect of commercial *T. harzianum* T-22 (KRL-AG2) in the management of tomato root rot caused by *R. solani*, and found that it could significantly reduce the disease incidence (from 50% to 17.2%). Moreover, the current studies mainly focus on screening biocontrol agents for controlling tomato root rot caused by *R. solani*, but do not deeply investigate the underlying molecular mechanisms.

In addition, several principal barriers limit the translation of biocontrol microorganisms from experimental success to reliable field performance and commercial implementation. The first is that the varying environmental conditions in the field cause the unstable performance of biocontrol microorganisms and lead to variable control efficacy. Second, formulations with long shelf lives and stable survival rates are lacking, which influences the commercial implementation of biocontrol agents.

For further effective control of tomato Rhizoctonia root rot through biocontrol agents, the following suggestions are made:

(1) Screen more biocontrol agents against tomato Rhizoctonia root rot. Currently, the number of biocontrol agents reported to be able to suppress tomato Rhizoctonia root remains limited. Multiple isolation and screening strategies should be used to identify more suitable biocontrol agents.
(2) Clearly identify the molecular mechanism of biocontrol agents in suppressing tomato Rhizoctonia root rot. By combining omics methods, including genomics, transcriptomics, proteomics, and metabolomics, a multi-level regulatory network can be constructed. An interaction network among biocontrol agents, *R. solani*, and tomato plants can be established, and then the molecular mechanisms of biocontrol agents in controlling tomato Rhizoctonia root rot can be comprehensively analyzed.
(3) Screen biocontrol-related genes. Biocontrol-related genes could be screened through multi-omics analysis, and their roles in controlling tomato Rhizoctonia root rot could be examined through gene function analyses, such as gene knockout, gene silencing, and gene overexpression analyses.
(4) Improve the control efficacy of biocontrol agents against tomato Rhizoctonia root rot. Numerous methods could be used to enhance the control efficacy of biocontrol agents. First, the culture and inoculation conditions of biocontrol agents should be optimized. Second, mutation breeding could be used to screen biocontrol agents highly effective in suppressing tomato Rhizoctonia root rot. Finally, effective biocontrol genes can be transferred into biocontrol agents to construct genetically modified strains, thereby improving the control efficacy of tomato Rhizoctonia root rot.
(5) Construct complex biocontrol microorganisms. The use of a single biocontrol microorganism can sometimes have limited effectiveness in controlling tomato Rhizoctonia root rot and may face challenges associated with multiple plant diseases. Therefore, different types of biocontrol agents, such as bacteria and fungi, as well as those from different environmental sources, such as endophytic microorganisms from various plants and soil-derived microorganisms, could be used to construct complex biocontrol microorganisms in order to achieve synergistic effects in disease control, plant growth promotion, stress resistance, and other multiple functions.
(6) Develop biocontrol commercial products. Currently, there are few commercial biocontrol products on the market for controlling tomato Rhizoctonia root rot. Biocontrol agents that exhibit excellent efficacy in controlling tomato Rhizoctonia root rot under field conditions could be used to develop more commercial products. Developing more commercial biocontrol products, as well as various formulations, and improving their stability are of great significance for the large-scale application of biocontrol agents to control tomato Rhizoctonia root rot.

## Data Availability

No new data were created or analyzed in this study. Data sharing is not applicable.
